# Intratumoral habitat combined with peritumoral radiomics to improve the predictive performance of initial TACE efficacy: a multicenter retrospective study

**DOI:** 10.3389/fonc.2026.1772206

**Published:** 2026-10-14

**Authors:** Zhefeng Wang, Ningbo Yin, Ruisi Su, Minhui Zhou, Zhichao Sun, Jiaying Li, Litian Chen

**Affiliations:** 1 Radiology Imaging Center, The First Affiliated Hospital of Wenzhou Medical University, Wenzhou, China; 2 The First Clinical Medical College of Zhejiang Chinese Medical University, Hangzhou, China; 3 Department of Radiology, The Central Affiliated Hospital of Shaoxing University (Shaoxing Central Hospital), Shaoxing, China; 4 Department of Radiology, The First Affiliated Hospital of Zhejiang Chinese Medical University (Zhejiang Provincial Hospital of Chinese Medicine), Hangzhou, China

**Keywords:** habitat imaging, hepatocellular carcinoma, prediction model, radiomics, transcatheter arterial chemoembolization

## Abstract

**Background and purpose:**

Transcatheter arterial chemoembolization (TACE) is the primary treatment for intermediate-stage hepatocellular carcinoma (HCC). However, significant heterogeneity in patient response to TACE necessitates non-invasive and precise methods for evaluating treatment efficacy. This study aims to explore the predictive ability of combined intratumoral habitat imaging and peritumoral radiomics features for TACE response.

**Materials and methods:**

We retrospectively enrolled HCC patients who underwent TACE treatment at four centers between January 2015 and December 2023. All patients underwent contrast-enhanced MRI. Patients from centers A and B formed the training cohort, while those from centers C and D comprised external validation cohorts. Using three-phase enhanced MRI (arterial, portal venous, and delayed phase), K-Means clustering divided intratumoral regions into three habitat subregions (Habitat 1, 2, and 3), and peritumoral features were extracted. Clinical variables were also collected. Key radiomics features were selected by LASSO regression, and clinical indicators were identified through univariable and multivariable logistic regression. The XGBoost algorithm constructed intratumoral, peritumoral, clinical, and combined models. Performance was evaluated using ROC curves, calibration curves, decision curve analysis (DCA), and DeLong tests.

**Results:**

A total of 174 HCC patients who underwent initial TACE treatment were enrolled, including 104 in training and 70 in validation cohorts. Fifteen key intratumoral features from the habitat subregions and 24 radiomics features from the peritumoral region were identified, primarily derived from wavelet transforms and texture analysis. The wavelet-LLL_glszm_ZoneVariance_ PVP feature from Cluster 2 contributed significantly to the model and had a positive impact (highest SHAP value). Alpha-fetoprotein (AFP) (P = 0.015) and Tumor size (P = 0.016) remained significantly associated with TACE treatment response in multivariable analysis. The combined model based on clinical, intratumoral habitat and peritumoral radiomics showed superior predictive performance, with AUCs of 0.950 (95% CI: 0.89-1.00), 0.850 (0.79-0.91) and 0.830 (0.77-0.89) in training and validation cohorts, outperforming single models (Clinic: AUC = 0.700, Tumor_Habitat: AUC = 0.880, Peritumor_Radiomics: AUC = 0.870, P<0.05). Calibration and DCA demonstrated good agreement between predicted and observed outcomes and favorable net benefit across the evaluated threshold probabilities.

**Conclusion:**

The combined model integrating AFP, Tumor size, intratumoral habitat imaging, and peritumoral radiomics could accurately predict initial TACE response in HCC patients. Cluster 2-derived features showed relatively greater contributions among the intratumoral habitat subregions. This non-invasive prediction tool may provide complementary information for pretreatment risk stratification. Future prospective multicenter studies are needed to verify the clinical applicability of the model.

## Introduction

Primary liver cancer represents a major global health burden, ranking as the sixth most prevalent malignant tumor worldwide and the third leading cause of cancer-related mortality (1). For patients with unresectable Barcelona Clinic Liver Cancer (BCLC) intermediate-stage hepatocellular carcinoma (HCC), transcatheter arterial chemoembolization (TACE) has emerged as the first-line therapeutic intervention (2). This locoregional treatment modality achieves its anti-tumor effects through dual mechanisms: occlusion of tumor vasculature and localized delivery of chemotherapeutic agents. While TACE has gained widespread clinical adoption, particularly in Asian populations (3, 4), substantial heterogeneity in treatment response remains a significant challenge, with a subset of patients demonstrating limited therapeutic benefit (5). Notably, early treatment response following initial TACE has been established as an independent prognostic factor for clinical outcomes, underscoring its critical role in guiding personalized treatment strategies for HCC patients (6, 7). Consequently, the development of robust early response prediction systems is paramount for optimizing therapeutic decision-making.

The complex biology of malignant tumors encompasses intricate initiation mechanisms and dynamic evolutionary processes. HCC exhibits marked intratumoral and peritumoral heterogeneity, with distinct microenvironmental characteristics that profoundly influence treatment response, disease progression, and recurrence patterns (8–10). The tumor microenvironment comprises two functionally interconnected yet distinct compartments: the intratumoral region, characterized by a complex interplay of neoplastic cells, immune infiltrates, vascular networks, and stromal components (11–13); and the peritumoral zone, which demonstrates active participation in inflammatory processes, fibrotic remodeling, and angiogenic responses (14–16). Emerging evidence has identified specific peritumoral imaging features (e.g., arterial phase enhancement, margin irregularity, and microsatellite distribution patterns) as reliable predictors of HCC recurrence (17–19). These observations highlight the pivotal role of both intratumoral and peritumoral microenvironments in determining TACE treatment outcomes.

Recent advances in radiomics have revolutionized tumor characterization by enabling non-invasive quantification of textural, morphological, and functional features (20). However, conventional radiomics approaches often treat tumors as homogeneous entities, potentially obscuring critical spatial heterogeneity (21). Habitat imaging represents a significant methodological advancement, allowing for tumor segmentation into discrete subregions with similar biological characteristics (22–24). This innovative approach facilitates the detection of subtle intratumoral variations while providing novel insights into tumor heterogeneity. The synergistic application of habitat imaging and radiomics offers a powerful framework for comprehensive spatial analysis of tumor heterogeneity and its relationship to therapeutic response.

While radiomics has shown promise in predicting HCC prognosis, most studies have focused on long-term outcomes such as recurrence and survival (25–27), whereas the preoperative prediction of initial response to TACE remains less well established. In addition, although intratumoral habitat imaging and peritumoral radiomics have been explored in HCC (28), few studies have specifically integrated training-cohort-derived intratumoral habitat analysis with a standardized intrahepatic peritumoral radiomics signature to predict initial TACE response. Such an integrated framework may better capture both intratumoral spatial heterogeneity and tumor-liver interface microenvironmental information, thereby providing additional value beyond conventional whole-tumor or single-region radiomics approaches. Therefore, this study aims to: (1) quantitatively characterize intratumoral habitat and peritumoral radiomics features in HCC using multiparametric MRI; (2) evaluate their association with initial TACE treatment response; and (3) develop an integrated predictive model to guide personalized therapeutic strategies.

## Materials and methods

### Patient characteristics

This study was approved by the institutional review board (No. 2025-KLS-219-01), and written informed consent was waived for this retrospective study. All research procedures involving human participants were conducted in strict compliance with the 1975 Helsinki Declaration and its subsequent amendments. The consecutive HCC patients enrolled between January 2015 and December 2023 met the following inclusion criteria: (a) age ≥18 years; (b) received TACE treatment; (c) liver function as Child-Pugh grade A or B; (d) Eastern Cooperative Oncology Group (ECOG) performance status score ranging from 0 to 2; (e) expected survival period > 3 months; (f) underwent contrast-enhanced MRI (CE-MRI) within one month before TACE. The diagnosis of HCC is either confirmed by histopathology or by non-invasive criteria defined by the American Association for the Study of Liver Diseases (AASLD), which is based on specific imaging features (29). The exclusion criteria were as follows: (a) previous treatment for HCC (e.g., TACE, ablation, chemotherapy, etc.); (b) concurrent other malignancies; (c) intraoperative simultaneous ablation procedure; (d) adjuvant treatment within 3 months post-TACE (e.g., immunotherapy, chemotherapy, etc.); (e) absence of short-term follow-up data; (f) poor image quality (**Figure 1**). Patients from centers A and B formed the training cohort, while patients from centers C and D formed the external validation cohorts.

**Figure 1:**
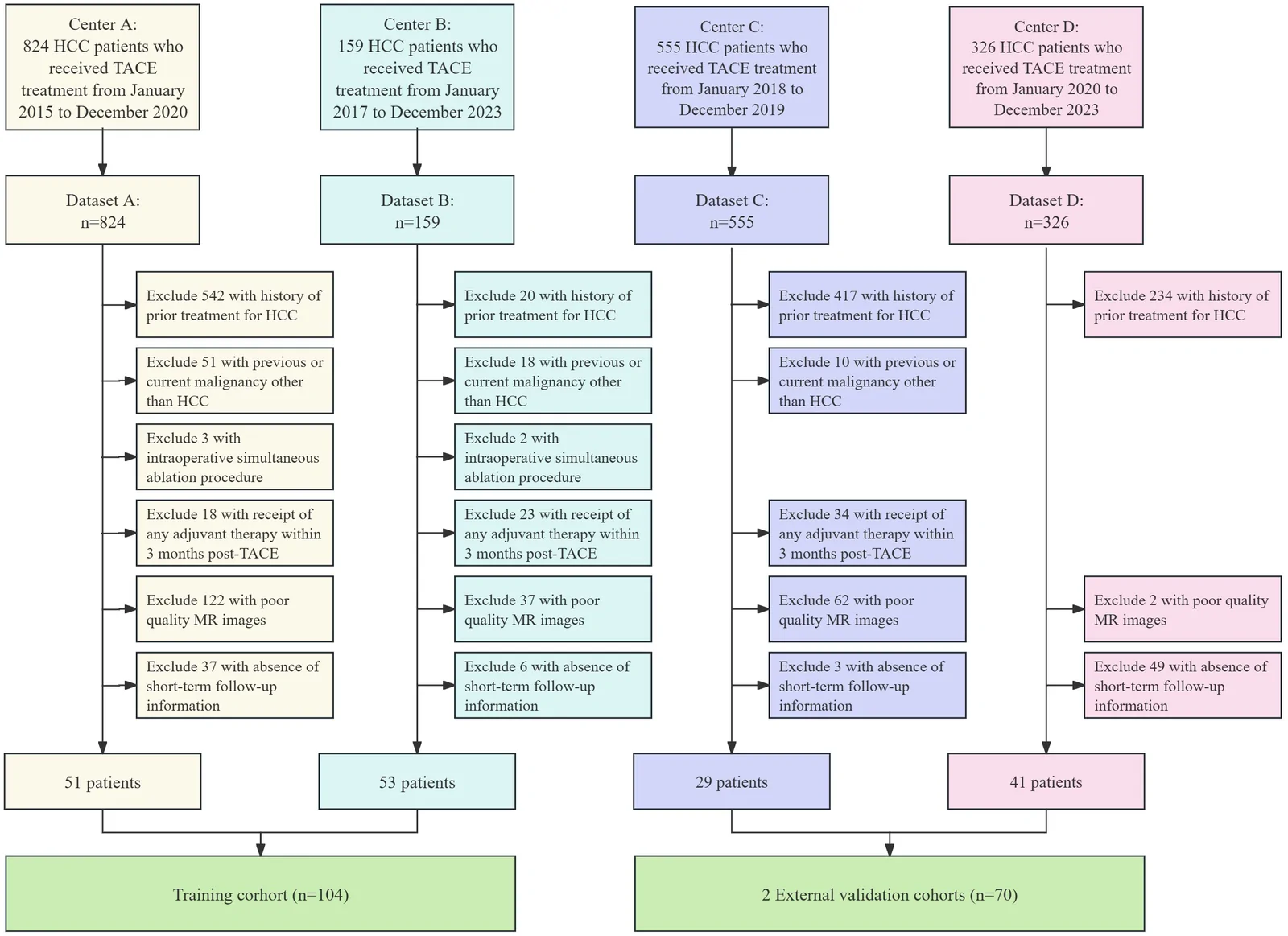
Flowchart shows patient exclusion for each data set.

### Baseline evaluation

Comprehensive clinical data were collected for all patients, including demographic characteristics (age and sex), virological history (hepatitis B or C infection), serum alpha-fetoprotein (AFP), total bilirubin (TBIL), albumin (ALB), prothrombin time (PT), alanine aminotransferase (ALT), aspartate aminotransferase (AST), and platelet count (PLT). Liver function was further stratified using the Child-Pugh and ALBI (Albumin-Bilirubin) grading systems, while tumor staging was classified according to the Barcelona Clinic Liver Cancer (BCLC) criteria. Additionally, patient performance status was evaluated using the ECOG (Eastern Cooperative Oncology Group) score.

### MR examination

MRI examinations were conducted using either a 1.5 T (Siemens Syngo MR B17) or 3.0 T scanner (GE Healthcare Discovery MR 750 or Siemens Verio MR), each equipped with a dedicated body array coil. The imaging protocol comprised T1-weighted (T1W), T2-weighted (T2W), diffusion-weighted imaging (DWI), and contrast-enhanced T1W sequences. For contrast-enhanced imaging, gadopentetate dimeglumine (Gd-DTPA) was administered intravenously via antecubital injection at a dose of 0.2 mL/kg and a flow rate of 2 mL/s. Enhanced phase imaging was performed with breath-holding at the following time points: arterial phase (25–35 s), portal venous phase (50–60 s), and delayed phase (150–240 s). Detailed acquisition parameters for all sequences are provided in **Supplementary Data S1**.

### Treatment procedure and outcome evaluation

TACE procedures were performed under the supervision of senior radiologists with over 10 years of clinical experience in interventional therapy. A detailed protocol of the TACE procedure is provided in **Supplementary Data S2**. TACE treatment response was evaluated using mRECIST criteria, with outcomes categorized as complete response (CR), partial response (PR), stable disease (SD), and progressive disease (PD). The objective response rate (ORR) represents the sum of CR and PR, while the non-response rate (NRR) includes SD and PD. Tumor response to treatment was evaluated by contrast-enhanced CT or MRI scan at 1–3 months following initial TACE. For patients with viable residual or recurrent tumors, salvage therapies, including repeat TACE, surgical resection, radiation therapy, or systemic treatments, were considered based on tumor characteristics and individual patient suitability.

### Tumor segmentation and habitat generation

Preoperative CE-MRI images (AP, PVP, and DP) were exported in DICOM format and loaded into the open-source software 3D Slicer (version 5.0.3; https://www.slicer.org) for 3D segmentation. All images were preprocessed (normalized, denoised, registered). At each cross section, the volumetric region of interest (VOI) was manually delineated layer by layer along the tumor contour as the intratumoral region of interest (VOI_intratumor). The 5-mm region from the surface of the tumor was defined as the peritumoral region (VOI_peritumor), which was segmented using a semi-automated approach (aided by the region growing algorithm in 3D Slicer). For cases where the tumor was adjacent to the liver capsule, only the intrahepatic parenchymal portion of the 5-mm peritumoral region was included, and extracapsular tissue was excluded. The 5-mm intrahepatic peritumoral margin was chosen to capture key pathological changes while minimizing normal tissue noise (30). If there were multiple lesions, the largest lesion was selected for segmentation. All VOIs were independently segmented by two abdominal radiologists with 7 and 10 years of experience, respectively. Inter-observer reproducibility was assessed using the intraclass correlation coefficient (ICC). An ICC > 0.75 was considered indicative of good reproducibility. The K-Means method was used to cluster voxel-wise multiphasic MRI signal-intensity vectors derived from the co-registered AP, PVP, and DP images within the VOI at the individual patient level to form multiple habitats. The optimal number of clusters was determined using the elbow method based on the sum of squared errors (SSE). Specifically, K values ranging from 2 to 10 were evaluated using the training cohort only, and the inflection point of the SSE curve indicated that K = 3 was optimal. The determined K = 3 was then fixed and applied to each patient in the training and two external validation cohorts without further optimization, resulting in three intratumoral habitat subregions (Habitat 1, Habitat 2, and Habitat 3). No validation cohort data were used to determine the optimal K value, thereby avoiding data leakage.


$$SSE=\sum_{i=1}^{\kappa }\sum_{x\in c_{i}}∥\chi -\mu_{i}{∥}^{2}$$

Note: *κ*: The preset total number of intratumoral habitat clusters to be partitioned. *i*: Index of each individual cluster, ranging from 1 to *κ*. *c_i_*: The *i*-th intratumoral habitat cluster containing a set of voxel-wise multiphasic intensity vectors. *χ*: A voxel-wise multiphasic MRI signal-intensity vector comprising the co-registered AP, PVP, and DP signal intensities at a given spatial location within the intratumoral VOI. *μ_i_*: The centroid (mean multiphasic intensity vector) of all voxels assigned to the *i*-th habitat cluster. $∥\chi -\mu_{i}{∥}^{2}$: Squared Euclidean distance between sample feature *χ* and its cluster centroid *μ_i_*.

### Feature extraction and selection

Pyradiomics software package (version 3.0.1, http://www.radiomics.io/pyradiomics.html) was used to extract the radiomic features from the intratumoral habitat subregions and peritumoral VOI. A total of 1,050 radiomics features were extracted from each region, including unfiltered original features (such as tumor intensity, texture, and shape), filtered image features (such as exponential filtering, gradient filtering, logarithm filtering, Laplacian of Gaussian filtering with sigma values of 1.0, 2.0 and 3.0, square filtering, square root filtering) and wavelet-based feature extraction methods. After removing outliers for all features using the 3-sigma rule, the data were normalized using the Z-score method, and then the independent-samples t-test or Mann-Whitney U test was used to screen for statistically significant features (P < 0.05). For each radiomic feature, values outside the range of mean ± 3 standard deviations were considered outliers and were capped at the corresponding lower or upper threshold. The mean and standard deviation used for outlier detection and Z-score normalization were calculated exclusively from the training cohort and subsequently applied unchanged to both external validation cohorts. For features with high repeatability, the Spearman rank correlation coefficient was used to calculate correlations between features, with |r| > 0.70 considered indicative of high correlation. Finally, the LASSO regression method was used to select the most relevant radiomic features. All feature selection procedures were performed using the training cohort only, and the selected features were subsequently applied unchanged to the external validation cohorts. The selected features were further interpreted using the Shapley Additive Explanations (SHAP) method, and the SHAP values were used to quantify the importance of each feature and its impact on the prediction probability of the model.

### Model construction and evaluation

The radscore was calculated based on the radiomics features selected from the intratumoral subregions and peritumoral region in the training cohort. Clinical and imaging variables were initially evaluated using univariable logistic regression in the training cohort, and variables with a two-sided P value < 0.05 were considered candidates for subsequent multivariable logistic regression analysis. Variables that did not meet this predefined screening criterion were not forcibly included in the multivariable model. The extreme gradient boosting (XGBoost) method was used to construct the intratumoral prediction model (Tumor, intratumoral habitat features only), peritumoral prediction model (Peritumor, peritumoral radiomics features only) and clinical prediction model (Clinic). In addition, to improve the predictive performance, the XGBoost method was further used to construct a combined model (Combined, intratumoral + peritumoral + clinical indicators). To facilitate clinical interpretation and individualized probability estimation, a nomogram was additionally constructed using the same predictors included in the Combined model. Specifically, these predictors were entered into a multivariable logistic regression model, and the resulting regression coefficients were used to construct the nomogram. The nomogram was intended as an auxiliary clinical visualization based on the same predictor set and was not directly derived from the internal parameters of the XGBoost model. The XGBoost models were trained in the training cohort and internally validated using 5-fold cross-validation, followed by further evaluation in the external validation cohorts. The final models were fitted using the entire training cohort, and the reported training cohort AUCs were calculated from these final models rather than from 5-fold cross-validation. The workflow for model construction is shown in **Figure 2**.

**Figure 2:**
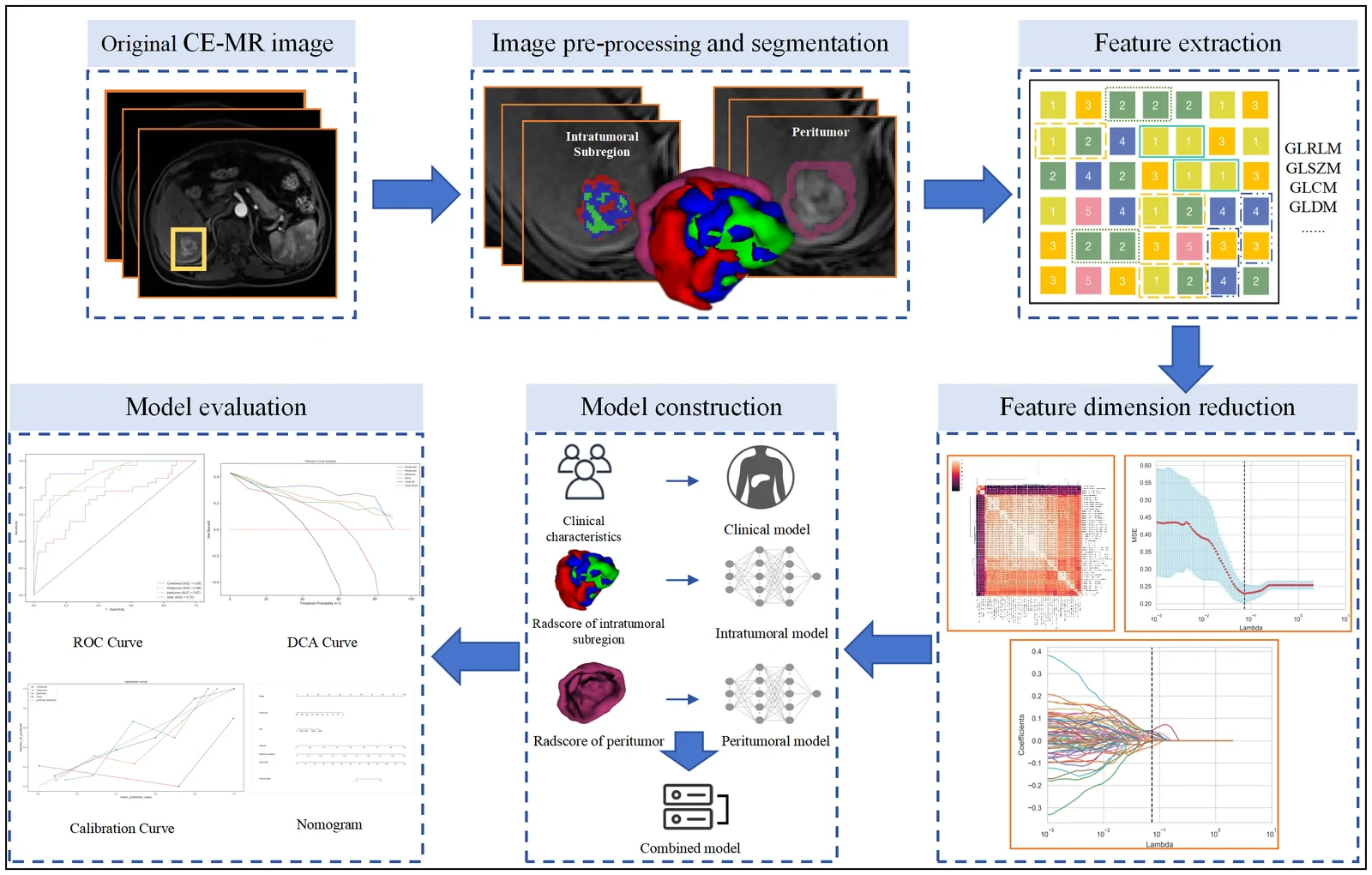
Schematic shows the workflow of this study.

### Statistical analysis

Python (version 3.7.6) was used for statistical analysis. Continuous normally distributed data were described by mean ± standard deviation (mean ± SD), and categorical data were described by frequency (percentage) (n (%)). Characteristics of continuous variables were compared using t-test or Mann-Whitney U-test, while categorical variables were compared using chi-square test or Fisher’s exact test. Univariable and multivariable logistic regression analysis were used to analyze the clinical indicators significantly related to tumor response. The performance of the prediction model was evaluated by receiver operating characteristic (ROC) curve analysis, and the area under the curve (AUC), accuracy, sensitivity, and specificity were obtained. Calibration curves were used to evaluate the consistency between the prediction accuracy of the model and the actual situation. The DeLong test compares the AUC of different models. Decision curve analysis (DCA) was used to estimate the clinical utility at different threshold probabilities. Statistical significance was defined as a two-sided P-value of <0.05.

## Results

### Patient characteristics

According to the screening criteria, a total of 174 HCC patients treated with TACE were finally enrolled in this study, with a mean age of (61.9 ± 12.0) years; of which 104 patients were included in the training cohort (Center A [n=51], Center B [n=53], the mean age was (62.7 ± 12.1) years), the ORR value was 56.7% (59/104), and the NRR value was 43.3% (45/104). The two external validation cohorts included 29 patients from Center C (mean age (57.2 ± 9.7) years; ORR = 58.6% (17/29), NRR = 41.3% (12/29)), 41 patients from Center D (mean age (63.0 ± 12.7) years; ORR = 39.1% (16/41), NRR = 60.9% (25/41). Significant intergroup differences (P < 0.05) were observed in AFP, total bilirubin, ALT, Child-Pugh grade, and BCLC stage among different cohorts (**Table 1**).

**Table 1:** Comparison of baseline characteristics between the training and external validation cohort.

Characteristic	Overall (n=174)	Training cohort (n=104)	External validation cohort C (n=29)	External validation cohort D (n=41)	*P* value
Age (y), mean (SD)	61.9 (12.0)	62.7 (12.1)	57.2 (9.7)	63.0 (12.7)	0.076
Sex, n (%)					0.407
Female	30 (17.2)	21 (20.2)	3 (10.3)	6 (14.6)	
Male	144 (82.8)	83 (79.8)	26 (89.7)	35 (85.4)	
Tumor number, n (%)					0.091
1	108 (62.1)	57 (54.8)	19 (65.5)	32 (78.0)	
2	15 (8.6)	12 (11.5)	1 (3.4)	2 (4.9)	
≥3	51 (29.3)	35 (33.7)	9 (31.0)	7 (17.1)	
Tumor size (mm), mean (SD)	69.7 (47.0)	68.2 (49.2)	75.3 (47.1)	69.5 (41.7)	0.775
Hepatitis, n (%)					0.660
HBV	126 (72.4)	74 (71.2)	23 (79.3)	29 (70.7)	
Non-HBV	48 (27.6)	30 (28.8)	6 (20.7)	12 (29.3)	
AFP (ng/ml), mean (SD)	4666.9 (12576.8)	2820.1 (6084.9)	9873.7 (23940.2)	5668.7 (12570.8)	0.023*
Albumin (g/L), mean (SD)	36.9 (5.9)	36.2 (6.3)	38.6 (5.3)	37.6 (5.0)	0.094
Total bilirubin (μmol/L), mean (SD)	18.2 (11.2)	20.7 (12.4)	14.0 (7.9)	15.0 (8.3)	0.002*
AST (U/L), mean (SD)	75.8 (164.8)	60.2 (82.6)	61.3 (29.7)	125.7 (309.7)	0.085
ALT (U/L), mean (SD)	55.7 (113.5)	41.4 (44.8)	50.9 (27.5)	95.4 (218.7)	0.034*
Cirrhosis, n (%)					0.430
Present	92 (52.9)	55 (52.9)	18 (62.1)	19 (46.3)	
Absent	82 (47.1)	49 (47.1)	11 (37.9)	22 (53.7)	
Child-Pugh grade, n (%)					0.010*
A	121 (69.5)	67 (64.4)	27 (93.1)	27 (65.9)	
B	53 (30.5)	37 (35.6)	2 (6.9)	14 (34.1)	
ALBI grade, n (%)					0.249
1	51 (29.3)	27 (26.0)	13 (44.8)	11 (26.8)	
2	114 (65.5)	71 (68.3)	16 (55.2)	27 (65.9)	
3	9 (5.2)	6 (5.8)		3 (7.3)	
BCLC stage, n (%)					0.001*
A	72 (41.4)	39 (37.5)	5 (17.2)	28 (68.3)	
B	32 (18.4)	20 (19.2)	8 (27.6)	4 (9.8)	
C	70 (40.2)	45 (43.3)	16 (55.2)	9 (22.0)	

AFP, Alpha-fetoprotein; ALBI, Albumin-bilirubin; ALT, Alanine aminotransferase; AST, Aspartate aminotransferase; BCLC, Barcelona clinic liver cancer; HBV, Hepatitis B virus; *Indicates statistical significance; *P* < 0.05.

### Variables associated with TACE treatment response in the training cohort

Univariable logistic regression analysis showed that AFP and tumor size were significantly associated with TACE treatment response (P < 0.05) and met the predefined criterion for subsequent multivariable analysis. Age was not significantly associated with treatment response (OR = 1.00, 95% CI: 0.96 - 1.05; P = 0.941). Multivariable logistic regression analysis further confirmed that Tumor size (P = 0.016) and AFP (P = 0.015) remained significantly associated with TACE treatment response (**Table 2**). Other evaluated clinical variables, including MRI type, hepatitis B virus infection status, cirrhosis status, and tumor number, did not meet the predefined significance criterion in the univariable analysis (P > 0.05).

**Table 2:** The association between treatment response and patient characteristics.

	Univariable analysis		Multivariable analysis	
Characteristic	Odds Ratio (95%CI)	*P* value	Odds ratio (95%CI)	*P* value
Age	1.00 (0.96-1.05)	0.941	–	–
Sex	0.53 (0.16-1.72)	0.291	–	–
MR type	1.86 (0.61-5.67)	0.277	–	–
Tumor number	0.74 (0.43-1.03)	0.264	–	–
Tumor size	1.02 (1.00-1.03)	0.022*	1.01 (1.00-1.04)	0.016^*^
Hepatitis	1.80 (0.65-5.02)	0.261	–	–
AFP	1.00 (1.00-1.00)	0.027*	1.10 (1.02-1.18)	0.015^*^
Albumin	0.93 (0.85-1.01)	0.084	–	–
Total bilirubin	1.01 (0.97-1.04)	0.753	–	–
AST	1.00 (0.99-1.01)	0.464	–	–
ALT	1.00 (0.99-1.02)	0.384	–	–
Cirrhosis	1.46 (0.57-3.74)	0.428	–	–
Child-Pugh grade	2.65 (0.98-7.17)	0.056	–	–
ALBI grade	1.88 (0.77-4.60)	0.166	–	–
BCLC stage	1.53 (0.90-2.60)	0.116	–	–

AFP, Alpha-fetoprotein; ALBI, Albumin-bilirubin; ALT, Alanine aminotransferase; AST, Aspartate aminotransferase; BCLC, Barcelona clinic liver cancer; HBV, Hepatitis B virus; MRI type, 1.5-T or 3.0-T MRI scanner; OR, Odds Ratio; CI, Confidence interval; The OR for AFP is reported per 1,000-ng/mL increase; *Indicates statistical significance; *P* < 0.05.

### Prognostic radiomic features from intratumoral habitats and peritumor region

All retained radiomic features met this criterion, with ICCs ranging from 0.83–0.90, 0.79–0.87, and 0.76–0.82 for AP, PVP, and DP images, respectively. After feature dimensionality reduction, a total of 15 key radiomic features were selected from the intratumoral habitat subregions and 24 from the peritumoral region,. These predictive features were mainly derived from different wavelet transforms and texture analysis. Among the 15 intratumoral habitat features, 5 were derived from Habitat 1, 6 from Habitat 2, and 4 from Habitat 3 (**Table 3**), indicating that predictive information was captured across all three habitat subregions. Feature-importance analysis further showed that Cluster 2-derived features had relatively greater contributions than those derived from Clusters 1 and 3 (**Figure 3a**). The peritumoral features covered a variety of texture and statistical characteristics and also showed substantial contributions to the prediction model (**Figure 3b**). SHAP analysis was further performed to illustrate the effects and relative contributions of individual radiomic features to the TACE response prediction model (**Figures 3c–f**). Among the intratumoral habitat features, wavelet-LLL_glszm_ZoneVariance_PVP_Cluster2 showed the greatest contribution to the model and had a positive impact on the prediction. The formula for calculating the radscore is provided in **Supplementary Data S3**.

**Table 3:** The prognostic radiomic features from intratumoral habitat.

Intratumoral subregion	Prognostic radiomic features
Cluster 1	wavelet-HLL_glszm_SmallAreaHighGrayLevelEmphasis_A
	wavelet-LLL_glszm_SmallAreaLowGrayLevelEmphasis_PVP
	wavelet-LHL_glszm_SmallAreaEmphasis_V
	original_gldm_DependenceVariance_V
	log-sigma-4-0-mm-3D_glszm_SmallAreaEmphasis_PVP
Cluster 2	wavelet-HHH_firstorder_Skewness_PVP
	wavelet-HLL_glcm_Imc1_PVP
	wavelet-HLH_glszm_SmallAreaLowGrayLevelEmphasis_A
	wavelet-LHH_glszm_SizeZoneNonUniformityNormalized_V
	wavelet-LHL_glszm_SmallAreaLowGrayLevelEmphasis_V
	wavelet-LLL_glszm_ZoneVariance_PVP
Cluster 3	wavelet-HLH_glszm_SmallAreaLowGrayLevelEmphasis_PVP
	wavelet-LLL_glszm_SmallAreaEmphasis_PVP
	log-sigma-4-0-mm-3D_glszm_SizeZoneNonUniformity_PVP
	log-sigma-3-0-mm-3D_glszm_SmallAreaLowGrayLevelEmphasis_V

**Figure 3:**
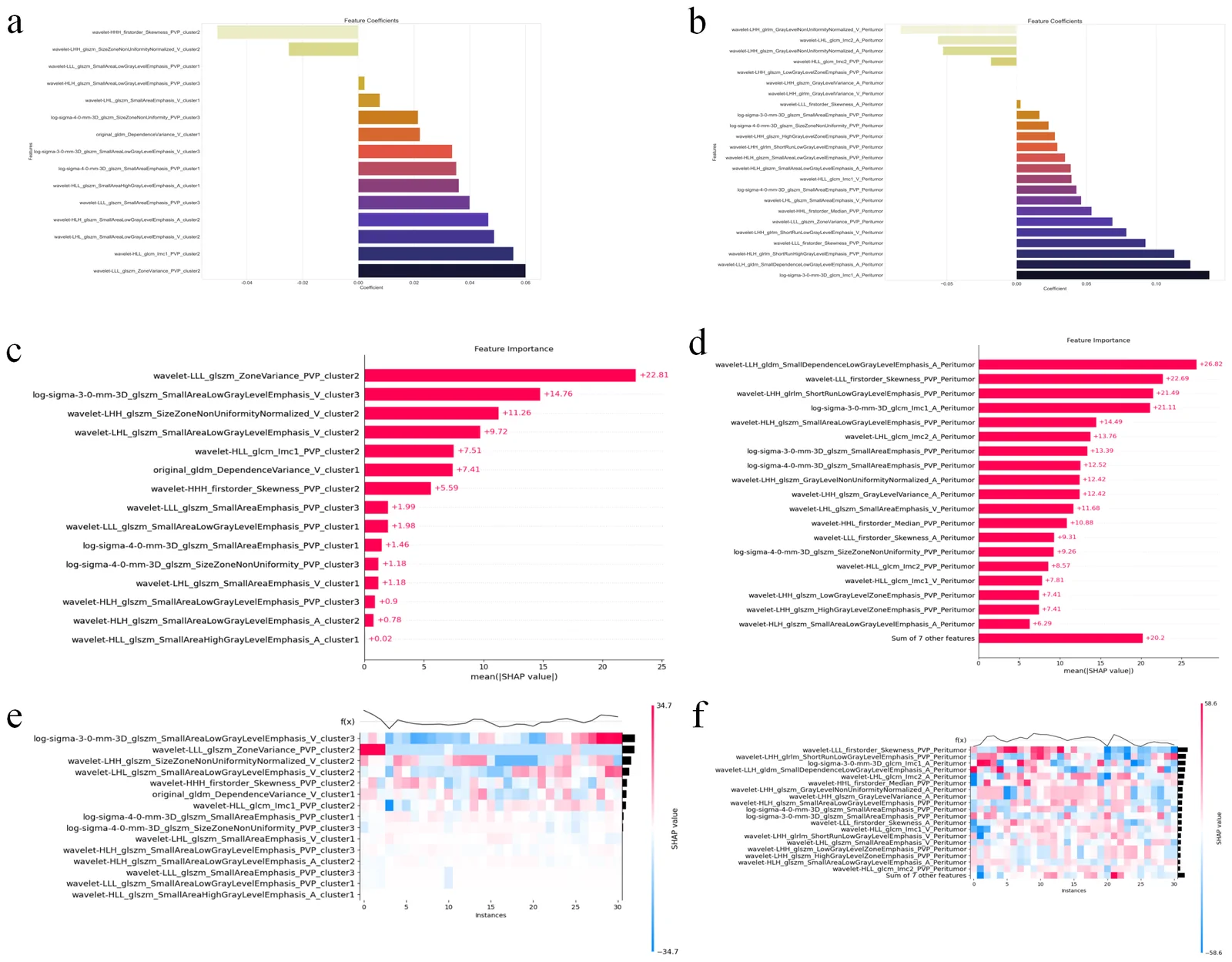
The feature importance of intratumoral habitat subregions **(a, c, e)** and peritumoral **(b, d, f)** radiomics features on the model and SHAP visualization. **(a, b)** Feature importance histogram: The vertical axis represents the 15 features selected, and the horizontal axis represents the feature importance coefficient of radiomics. **(c, d)** mean (| SHAP value |) bar chart. The horizontal axis is mean (| SHAP value |), and the larger the value, the more significant the overall impact of the feature on the model. The vertical axis is the ranking of features in descending order of importance. **(e, f)** Beeswarm plot. The horizontal axis is the SHAP value, and the positive values are the feature that has a positive contribution to the model, which increase the prediction probability of the model to the ORR. Negative values are negative contributions, which reduce the prediction probability of the model for the ORR. The vertical axis is the key prognostic features screened. Each point in the plot represents a sample, red (High) indicates high eigenvalue, blue (Low) indicates low eigenvalue, and the distribution of points indicates how much they impact the final result.

### Performance evaluation of prediction model

The AUC values reported for the training cohort were calculated from the final models fitted using the entire training cohort and did not represent the 5-fold cross-validation AUCs. The AUC values of the Clinic, Tumor, Peritumor and Combined in the training cohort were 0.70 (95% CI: 0.64-0.76), 0.87 (95% CI: 0.81-0.93), 0.88 (95% CI: 0.82-0.94), and 0.95 (95% CI: 0.89-1.00), respectively (**Figures 4a–c**; **Table 4**). The DeLong test further showed that the Combined model significantly outperformed the Clinic, Intratumor, and Peritumor models (P = 0.022, 0.032, and 0.025, respectively). The Intratumor model also significantly outperformed the Peritumor and Clinic models (P = 0.041 and 0.005, respectively), while the Peritumor model significantly outperformed the Clinic model (P = 0.021). These findings indicate that the intratumoral habitat-based model provided greater discriminative performance than the clinical and peritumoral models, while further integration of intratumoral habitat, peritumoral radiomic, and clinical information achieved the highest predictive performance. In the two external validation cohorts, the Combined model achieved AUCs of 0.85 (95% CI: 0.79-0.91) and 0.83 (95% CI: 0.77-0.89), respectively (**Table 4**), and maintained better predictive performance than the other models. The calibration curves showed good agreement between the predicted probabilities and observed outcomes for the Combined model in the training and external validation cohorts (**Figures 4d–f**). DCA further showed that the Combined model provided greater clinical net benefit than the individual models across the evaluated threshold probabilities (**Figures 4g–i**).

**Figure 4:**
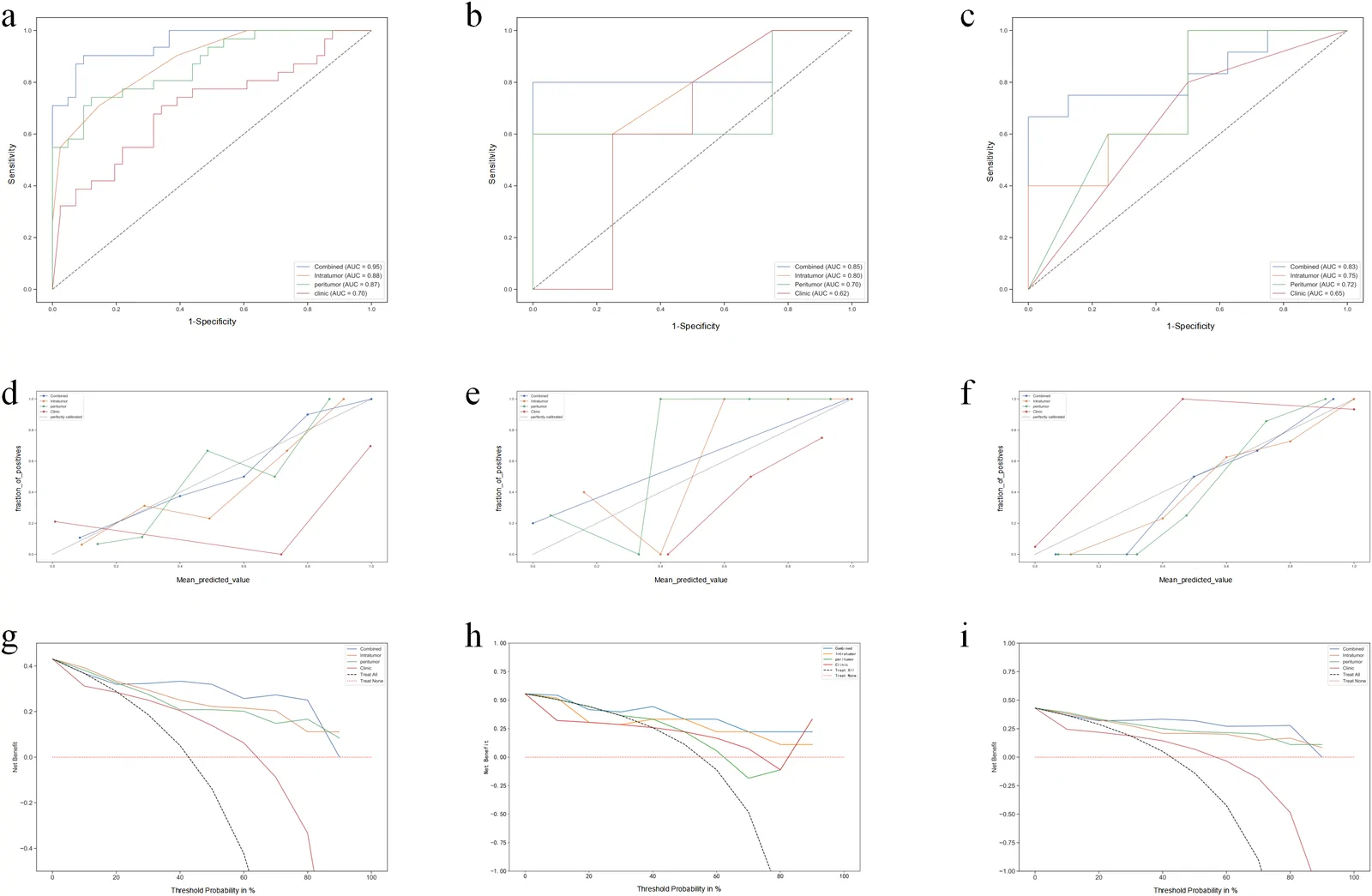
The ROC curves, calibration curves, and DCA curves for all models in training cohort **(a, d, g)**, validation cohort 1 **(b, e, h)**, and validation cohort 2 **(c, f, i)**. **(a–e)** ROC curves for different models. **(d–f)** The calibration curves show good consistency between the predicted and the actual values of the Combined. **(g–i)** The DCA curves show that the Combined has a strong predictive ability of the TACE response of HCC in a specific probability range, providing a good clinical benefit.

**Table 4:** Prediction performance of final models in training cohort and validation cohorts.

Cohort	Model	AUC	95%Cl	Accuracy	Sensitivity	Specificity	Precision	PPV	NPV
Train	Combined	0.950	0.89-1.00	0.850	0.750	0.820	0.840	0.829	0.827
	Intratumor	0.880	0.82-0.94	0.820	0.783	0.804	0.816	0.767	0.762
	Peritumor	0.870	0.81-0.93	0.750	0.733	0.750	0.809	0.724	0.748
	Clinic	0.700	0.64- 0.76	0.730	0.667	0.720	0.789	0.703	0.736
Test 1	Combined	0.850	0.79-0.91	0.778	0.760	0.780	0.787	0.729	0.767
	Intratumor	0.800	0.74-0.86	0.725	0.690	0.750	0.742	0.686	0.718
	Peritumor	0.700	0.64-0.76	0.706	0.680	0.725	0.714	0.675	0.715
	Clinic	0.620	0.56-0.68	0.678	0.650	0.696	0.692	0.629	0.667
Test 2	Combined	0.830	0.77-0.89	0.801	0.886	0.857	0.839	0.832	0.892
	Intratumor	0.750	0.69-0.81	0.789	0.867	0.829	0.824	0.743	0.864
	Peritumor	0.720	0.66-0.78	0.769	0.819	0.782	0.803	0.732	0.827
	Clinic	0.650	0.59-0.71	0.718	0.791	0.758	0.701	0.716	0.787

The AUC values reported for the training cohort were calculated from the final models fitted using the entire training cohort and did not represent the 5-fold cross-validation AUCs. AUC, Area under the curve; CI, Confidence interval; PPV, Positive Predictive Value; NPV, Negative Predictive Value.

### Important intratumoral habitation features and example model interpretation

For clinical visualization, a logistic regression-based nomogram was additionally constructed using the same four predictors included in the Combined model to facilitate individualized estimation of the probability of non-response after initial TACE treatment in patients with HCC (**Figure 5**). **Figure 6** shows two HCC patients who were treated with TACE but with different treatment outcomes. According to the nomogram of the Combined in this study, there was a 30% probability that the post-treatment response of patient A would be NRR and the actual treatment response was PR. There was a 65% probability that the post-treatment response of patient B would be NRR, and the actual treatment response was SD.

**Figure 5:**
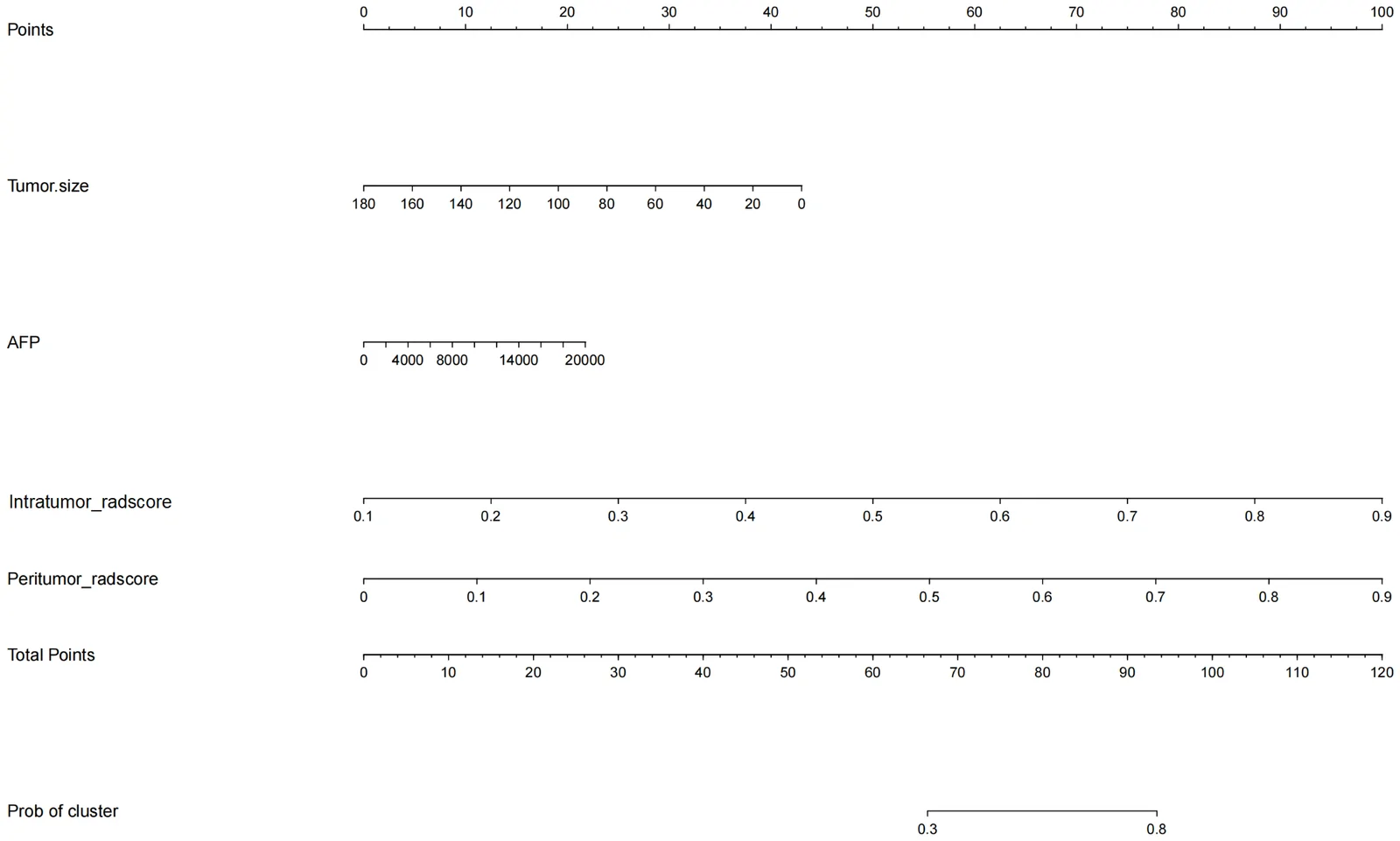
Nomogram for individualized prediction of non-response to initial TACE in patients with HCC. The nomogram was constructed based on four predictors: AFP, tumor size, intratumoral radscore, and peritumoral radscore. Each predictor is assigned a corresponding number of points, and the total points are mapped to the estimated probability of non-response to initial TACE. AFP, alpha-fetoprotein; HCC, hepatocellular carcinoma; TACE, transcatheter arterial chemoembolization.

**Figure 6:**
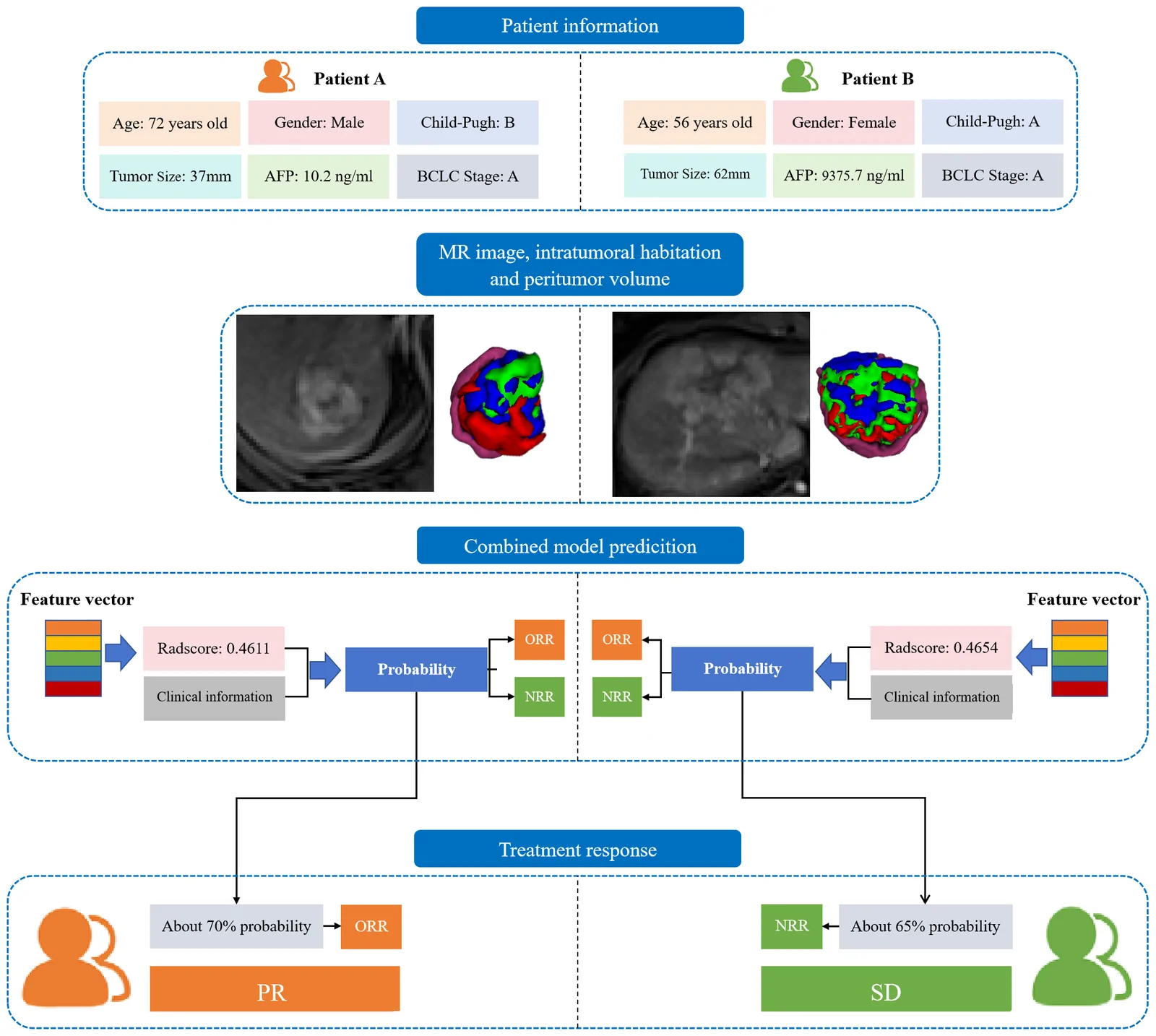
Representative clinical application of the prediction model in two patients with HCC undergoing initial TACE. Preoperative clinical information and contrast-enhanced MRI were used to obtain intratumoral habitat and peritumoral radiomic features. The corresponding radscores, together with AFP and tumor size, were incorporated into the prediction model to estimate the probability of non-response to initial TACE. Patient A had a predicted non-response probability of 30% and achieved PR, whereas patient B had a predicted non-response probability of 65% and had SD after treatment. AFP, alpha-fetoprotein; HCC, hepatocellular carcinoma; MRI, magnetic resonance imaging; PR, partial response; SD, stable disease; TACE, transcatheter arterial chemoembolization.

## Discussion

Non-invasive imaging technology plays a critical role in predicting the survival and treatment response of HCC patients. However, ideal models capable of accurately predicting the efficacy of initial TACE treatment remain scarce. In this study, we developed and validated a multimodal radiomics model integrating intratumoral habitat, peritumoral features, and clinical indicators (AFP and tumor size) to predict the response to initial TACE in patients with HCC. The Combined model achieved an AUC of 0.95 in the training cohort and maintained predictive performance in the two external validation cohorts, with AUCs of 0.85 and 0.83, respectively. Moreover, the Combined model showed better predictive performance than the Clinic, Tumor, and Peritumor models. Specifically, 15 predictive radiomic features were selected from the three intratumoral habitat subregions and 24 from peritumoral region, with Cluster 2-derived features showing relatively greater contributions among the intratumoral habitat subregions. These findings support the complementary value of intratumoral habitat and peritumoral radiomic information for predicting initial TACE response.

Previous studies have demonstrated that combined intratumoral and peritumoral radiomic features have potential value in evaluating and predicting outcomes after TACE in patients with HCC (29). Studies integrating radiomic features with clinical indicators have also shown promising performance in predicting treatment response (31), recurrence (32), and overall survival (33). However, most of these studies analyzed the tumor as a whole and therefore may not fully account for intratumoral spatial heterogeneity. Given the substantial heterogeneity in the response of HCC to TACE (34), the present study divided the tumor into three habitat subregions and constructed prediction models using radiomic features extracted from these subregions. Our results showed that the Intratumor model outperformed the Clinic and Peritumor models, while integration of intratumoral, peritumoral, and clinical information in the Combined model further improved predictive performance. These findings are consistent with previous studies. Wang et al. (35) reported that a model based on intratumoral habitat radiomic features outperformed a conventional radiomics model. Similarly, Wu et al. (36) demonstrated the potential value of radiomic features derived from different intratumoral subregions for characterizing tumor heterogeneity. Taken together, our findings suggest that habitat-based characterization of intratumoral heterogeneity may provide complementary predictive information for TACE response, while integration with peritumoral and clinical information may further improve model performance.

In this study, we found that the predictive radiomic features of the intratumoral habitats and peritumoral region were mainly derived from different wavelet transforms and texture feature analyses. Among them, the predictive features derived from the intratumoral habitat subregions showed similarities in feature composition and were predominantly related to the quantification of tumor heterogeneity, structural complexity, and gray-level distribution, which have been associated with tumor invasion, prognosis, and treatment response (37–39), including GLSZM-, GLDM-, and GLCM-derived features. The features derived from the Cluster 2 subregion showed relatively greater contributions among the intratumoral habitat subregions, and they were all wavelet-derived features, which can quantify texture heterogeneity, gray-level distribution, and structural complexity in tumor imaging (40). Notably, multiple features related to Small Area Low Gray Level Emphasis were selected and were mainly concentrated in Cluster 2. These features reflected the presence of a relatively large number of small areas with low gray values within the tumor, which may be associated with pathological heterogeneity, such as fibrosis or necrosis, as suggested by previous studies (41, 42). Such tumor microenvironmental heterogeneity may contribute to a poor response to TACE (43). In addition, features such as Small Area High Gray Level Emphasis, Median, and High Gray Level Zone Emphasis may be associated with tumor proliferative activity (44–46). These findings suggest that the selected features within individual habitat subregions captured related aspects of intratumoral heterogeneity, including gray-level distribution and texture complexity. Partitioning the tumor into distinct habitat subregions may therefore facilitate more detailed characterization of spatial heterogeneity and subtle imaging variations within the tumor (47).

This study also found that peritumoral features contributed substantially to the model and provided complementary information for predicting TACE treatment response. Specifically, Gray Level Non-Uniformity Normalized features characterize variations in gray-level distribution within the peritumoral region, while Imc2 (Informational Measure of Correlation 2) features describe texture complexity and dependency. In addition, the peritumoral first-order feature Skewness characterizes asymmetry in the gray-level intensity distribution. Previous studies have suggested that such peritumoral imaging characteristics may be associated with tumor aggressiveness and invasive biological behavior, including microvascular invasion (48). The peritumoral region plays an important role in tumor recurrence, invasion, and metastasis and has potential value for improving prediction model performance (49). However, previous studies have suggested that its heterogeneity may be lower and its radiomic characteristics less pronounced than those of the tumor core region (50–53). Based on these considerations, the peritumoral region was not further divided into habitat subregions in this study, and the potential value of peritumoral habitat analysis requires further investigation in larger cohorts. Nevertheless, the contribution of peritumoral features observed in our study, together with the improved performance of the Combined model, suggests that peritumoral radiomic information may provide complementary predictive information beyond intratumoral habitat features. Integrating intratumoral habitat, peritumoral radiomics, and clinical information may therefore enable more comprehensive characterization of tumor imaging heterogeneity and improve the prediction of TACE treatment response.

In the construction of the clinical model, we evaluated potential clinical variables and found that AFP and tumor size were significantly associated with TACE treatment response and remained significant in the multivariable analysis; therefore, these two variables were included in the clinical model. AFP is a tumor marker widely used in the clinical evaluation of HCC, and elevated AFP levels have been associated with tumor progression and poorer treatment outcomes (54). Previous studies have also reported an association between higher AFP levels and poorer response to TACE (55, 56), consistent with the association observed in our cohort. Tumor size was also significantly associated with treatment response in our study. Larger tumors generally represent a greater tumor burden and have been associated with more aggressive tumor characteristics and poorer outcomes after TACE (2, 57). These findings are consistent with previous studies suggesting that AFP and tumor size may provide clinically relevant information for evaluating TACE treatment response (58). In the present study, however, the predictive performance of the Clinic model was lower than that of the radiomics-based models, whereas integration of AFP and tumor size with intratumoral habitat and peritumoral radiomic information further improved predictive performance. These results suggest that clinical and radiomic information may provide complementary value for predicting TACE treatment response. To facilitate individualized probability estimation, a nomogram was additionally constructed using the same four predictors included in the Combined model (AFP, tumor size, intratumoral radscore, and peritumoral radscore). The non-invasive nomogram provides an intuitive visualization of the estimated probability of non-response to initial TACE. However, its potential clinical utility requires further prospective validation.

This study used triphasic contrast-enhanced MRI (AP, PVP, and VP) for radiomic feature extraction and prediction of TACE treatment response. The selected predictive features were derived from different contrast-enhanced phases, suggesting that multiphasic MRI provides complementary imaging information for characterizing tumor heterogeneity. Previous studies have shown that contrast-enhanced MRI can provide information related to tumor vascularity, perfusion characteristics, and tissue composition, which may be relevant to TACE treatment response (59). In the present study, radiomic features derived from AP, PVP, and DP images contributed to the prediction models, supporting the value of integrating multiphasic imaging information rather than relying on a single phase. MRI examinations in this multicenter study were acquired using different scanners and magnetic field strengths, reflecting the heterogeneity of imaging conditions encountered in clinical practice. Previous studies have suggested that radiomic models may maintain predictive performance across different MRI scanners and magnetic field strengths (60). However, scanner-specific subgroup analysis was not performed in the present study. Therefore, the potential influence of scanner-related variability cannot be fully excluded and should be further evaluated in larger prospective multicenter studies. Overall, our findings support the potential value of triphasic contrast-enhanced MRI radiomics for predicting response to initial TACE in patients with HCC.

Notably, the combined model demonstrates robust predictive performance, achieving an AUC of 0.950 in the training cohort and maintaining stability across two independent external validation cohorts, significantly outperforming conventional clinical parameters alone (AUC = 0.70). This non-invasive MRI-based nomogram serves as a practical tool for preoperative risk stratification in intermediate-stage HCC patients undergoing initial TACE. Clinically, it facilitates individualized decision-making by quantifying the probability of non-response: low-risk patients may be prioritized for conventional TACE, whereas high-risk patients may benefit from combination therapies (e.g., targeted or immunotherapy). Furthermore, because it relies solely on routine contrast-enhanced MRI, it provides a safe alternative to percutaneous biopsy for patients with contraindications. From a translational perspective, the model provides a non-invasive approach for pre-treatment prediction of response to initial TACE by integrating intratumoral habitat, peritumoral radiomic, and clinical information. Its performance in two external validation cohorts further supports its potential for pre-treatment risk stratification. However, prospective studies are required to determine whether model-guided risk stratification can improve treatment planning and clinical outcomes. Ultimately, this framework integrates macroscopic clinical characteristics with intratumoral spatial heterogeneity and provides a potential approach for individualized pre-treatment assessment of TACE response.

However, this study still has some limitations. First, this study adopts a retrospective design, which may introduce selection bias and data incompleteness and further compromise the generalization ability of these models. Therefore, the clinical applicability and stability of these models need to be further verified through prospective studies with multicenter and large samples in the future. Second, significant differences in baseline (including AFP, total bilirubin, ALT, Child-Pugh grade, and BCLC stage) were observed between the training and validation cohorts, reflecting real-world clinical heterogeneity. Similar baseline imbalances between training and validation cohorts have also been reported in previous HCC prognostic model studies (61). While such disparities may theoretically limit the model’s external generalizability, propensity score matching (PSM) was not applied. Applying PSM would alter natural patient distributions and compromise the authenticity of external validation, especially given the relatively small validation sample sizes (62). Notably, despite this inherent heterogeneity, our combined model maintained stable predictive performance across both validation cohorts, substantiating its robustness in diverse clinical settings. Third, although TACE followed standardized protocols, minor inter-center differences in chemotherapeutic agents, embolic materials, and dosing existed per local practice. Core goals (complete embolization) and endpoints (reduced tumor blood flow/staining) were unified to mitigate confounding. This procedural variability is an inherent limitation, and future studies will adopt fully standardized TACE protocols. Fourth, the sample size of each cohort in this study is relatively limited, and future studies should further expand the sample size, especially to include more heterogeneous populations, so as to enhance the reliability and universality of the study conclusions. Fifth, manual segmentation of intratumoral and peritumoral regions was susceptible to subjectivity and inter-operator variability. Sixth, MRI data in this study were acquired using scanners from different vendors with 1.5T and 3.0T field strengths, and no specialized harmonization techniques were applied to alleviate batch effects. The heterogeneities in scanner hardware and field strengths may introduce confounding bias into image texture features. This issue will be resolved by implementing unified imaging protocols and standardized preprocessing pipelines in subsequent investigations. In the future, automated segmentation methods based on deep learning can be explored to improve segmentation efficiency, consistency, and repeatability. Finally, this study did not incorporate potential clinical and biological markers (e.g., genomic markers, epigenetic profiles). Integrating liquid biopsy, multi-omics data (e.g., transcriptomics, proteomics), and molecular pathological information in future studies may further optimize model performance, enhance biological interpretability, and provide more comprehensive support for personalized treatment decisions.

## Conclusion

In conclusion, this study proposes an innovative strategy for the precise and individualized management of HCC patients. We constructed a new comprehensive prediction model that integrated clinical indicators (AFP, Tumor size) and intratumoral habitat features and peritumoral radiomics features based on three-phase enhanced MRI. Among them, the Cluster2 subregion may be a key region affecting the poor response to TACE treatment. In addition, the non-invasive nomogram developed based on the combined model can accurately predict the tumor response of HCC patients after initial TACE treatment, which provides an important theoretical basis and clinical reference for individualized treatment decisions. Notably, subsequent prospective studies should be conducted to confirm the long-term stability of the model, ensuring its sustained validity in long-term follow-up and broader clinical promotion.

## Data Availability

The raw data supporting the conclusions of this article will be made available by the authors, without undue reservation.

## References

[1] Sung H Ferlay J Siegel RL Laversanne M Soerjomataram I Jemal A . Global cancer statistics 2020: GLOBOCAN estimates of incidence and mortality worldwide for 36 cancers in 185 countries. CA Cancer J Clin. (2021) 71:209–49. doi: 10.3322/caac.21660 33538338

[2] Reig M Forner A Rimola J Ferrer-Fábrega J Burrel M Garcia-Criado Á . BCLC strategy for prognosis prediction and treatment recommendation: The 2022 update. J Hepatol. (2022) 76:681–93. doi: 10.1016/j.jhep.2021.11.018 34801630 PMC8866082

[3] Goh EC Jeong-Hoon L Kim HY Hwang SY Kim JS Chung JW . Transarterial chemoembolization can be safely performed in patients with hepatocellular carcinoma invading the main portal vein and may improve the overall survival. Radiology. (2011) 258:627–34. doi: 10.1148/radiol.10101058 21273524

[4] Yau T Tang VY Yao TJ Fan ST Lo CM Poon RT . Development of Hong Kong Liver Cancer staging system with treatment stratification for patients with hepatocellular carcinoma. Gastroenterology. (2014) 146:1691–1700.e3. doi: 10.1053/j.gastro.2014.02.032 24583061

[5] Kudo M Kawamura Y Hasegawa K Tateishi R Kariyama K Shiina S . Management of hepatocellular carcinoma in Japan: JSH consensus statements and recommendations 2021 update. Liver Cancer. (2021) 10:181–223. doi: 10.1159/000514174 34239808 PMC8237791

[6] Han G Berhane S Toyoda H Bettinger D Elshaarawy O Chan AWH . Prediction of survival among patients receiving transarterial chemoembolization for hepatocellular carcinoma: A response-based approach. Hepatology. (2020) 72:198–212. doi: 10.1002/hep.31022 31698504 PMC7496334

[7] Adhoute X Penaranda G Naude S Raoul JL Perrier H Bayle O . Retreatment with TACE: the ABCR SCORE, an aid to the decision-making process. J Hepatol. (2015) 62:855–62. doi: 10.1016/j.jhep.2014.11.014 25463541

[8] Friemel J Frick L Weber A . Intratumor heterogeneity in HCC. Aging (Albany NY). (2015) 7:350–1. doi: 10.18632/aging.100760 26081519 PMC4505157

[9] Dagogo-Jack I Shaw AT . Tumor heterogeneity and resistance to cancer therapies. Nat Rev Clin Oncol. (2018) 15:81–94. doi: 10.1038/nrclinonc.2017.166 29115304

[10] Biswas A De S . Drivers of dynamic intratumor heterogeneity and phenotypic plasticity. Am J Physiol Cell Physiol. (2021) 320:C750–60. doi: 10.1152/ajpcell.00575.2020 33657326 PMC8163571

[11] de Visser KE Joyce JA . The evolving tumor microenvironment: From cancer initiation to metastatic outgrowth. Cancer Cell. (2023) 41:374–403. doi: 10.1016/j.ccell.2023.02.016 36917948

[12] Zhao Y Shen M Wu L Yang H Yao Y Yang Q . Stromal cells in the tumor microenvironment: accomplices of tumor progression? Cell Death Dis. (2023) 14:587. doi: 10.1038/s41419-023-06110-6 37666813 PMC10477351

[13] Grasmann G Mondal A Leithner K . Flexibility and adaptation of cancer cells in a heterogenous metabolic microenvironment. Int J Mol Sci. (2021) 22:1476. doi: 10.3390/ijms22031476 33540663 PMC7867260

[14] Liu W Gao L Hou X Feng S Yan H Pan H . TWEAK signaling-induced ID1 expression drives malignant transformation of hepatic progenitor cells during hepatocarcinogenesis. Adv Sci (Weinh). (2023) 10:e2300350. doi: 10.1002/advs.202300350 37085918 PMC10288241

[15] Liu J Chen S Wang W Ning BF Chen F Shen W . Cancer-associated fibroblasts promote hepatocellular carcinoma metastasis through chemokine-activated hedgehog and TGF-β pathways. Cancer Lett. (2016) 379:49–59. doi: 10.1016/j.canlet.2016.05.022 27216982

[16] Gu Y Guo Y Gao N Fang Y Xu C Hu G . The proteomic characterization of the peritumor microenvironment in human hepatocellular carcinoma. Oncogene. (2022) 41:2480–91. doi: 10.1038/s41388-022-02264-3 35314790 PMC9033583

[17] An C Kim DW Park YN Chung YE Rhee H Kim MJ . Single hepatocellular carcinoma: Preoperative MR imaging to predict early recurrence after curative resection. Radiology. (2015) 276:433–43. doi: 10.1148/radiol.15142394 25751229

[18] Lee S Kim SH Lee JE Sinn DH Park CK . Preoperative gadoxetic acid-enhanced MRI for predicting microvascular invasion in patients with single hepatocellular carcinoma. J Hepatol. (2017) 67:526–34. doi: 10.1016/j.jhep.2017.04.024 28483680

[19] Kim S Shin J Kim DY Choi GH Kim MJ Choi JY . Radiomics on gadoxetic acid-enhanced magnetic resonance imaging for prediction of postoperative early and late recurrence of single hepatocellular carcinoma. Clin Cancer Res. (2019) 25:3847–55. doi: 10.1158/1078-0432.CCR-18-2861 30808773

[20] Lambin P Leijenaar RTH Deist TM Peerlings J de Jong EEC van Timmeren J . Radiomics: the bridge between medical imaging and personalized medicine. Nat Rev Clin Oncol. (2017) 14:749–62. doi: 10.1038/nrclinonc.2017.141 28975929

[21] Bo Z Song J He Q Chen B Chen Z Xie X . Application of artificial intelligence radiomics in the diagnosis, treatment, and prognosis of hepatocellular carcinoma. Comput Biol Med. (2024) 173:108337. doi: 10.1016/j.compbiomed.2024.108337 38547656

[22] Chang YC-C Ackerstaff E Tschudi Y Jimenez B Foltz W Fisher C . Delineation of tumor habitats based on dynamic contrast enhanced MRI. Sci Rep. (2017) 7:9746. doi: 10.1038/s41598-017-09932-5 28851989 PMC5575347

[23] Zhang X Su GH Chen Y Gu YJ You C . Decoding intratumoral heterogeneity: Clinical potential of habitat imaging based on radiomics. Radiology. (2023) 309:e232047. doi: 10.1148/radiol.232047 38085080

[24] O'Connor JP Rose CJ Waterton JC Carano RA Parker GJ Jackson A . Imaging intratumor heterogeneity: role in therapy response, resistance, and clinical outcome. Clin Cancer Res. (2015) 21:249–57. doi: 10.1158/1078-0432.CCR-14-0990 25421725 PMC4688961

[25] Chong HH Yang L Sheng RF Yu Y-L Wu DJ Rao S-X . Multi-scale and multi-parametric radiomics of gadoxetate disodium-enhanced MRI predicts microvascular invasion and outcome in patients with solitary hepatocellular carcinoma ≤ 5 cm. Eur Radio. (2021) 31:4824–38. doi: 10.1007/s00330-020-07601-2 33447861 PMC8213553

[26] Chen K Sui C Wang Z Liu Z Qi L Li X . Habitat radiomics based on CT images to predict survival and immune status in hepatocellular carcinoma, a multi-cohort validation study. Transl Oncol. (2025) 52:102260. doi: 10.1016/j.tranon.2024.102260 39752907 PMC11754828

[27] Liu H-F Wang M Wang Q Lu Y Lu Y-J Sheng Y . Multiparametric MRI-based intratumoral and peritumoral radiomics for predicting the pathological differentiation of hepatocellular carcinoma. Insights into Imaging. (2024) 15(2024):97. doi: 10.1186/s13244-024-01623-w 38536542 PMC10973314

[28] Li J Zhou M Tong Y Chen H Su R Tao Y . Tumor growth pattern and intra- and peritumoral radiomics combined for prediction of initial TACE outcome in patients with primary hepatocellular carcinoma. J Hepatocellular Carcinoma. (2024) 11:1927–44. doi: 10.2147/JHC.S480554 39398867 PMC11471153

[29] Zhao Y Zhang J Wang N Xu Q Liu Y Liu J . Intratumoral and peritumoral radiomics based on contrast-enhanced MRI for preoperatively predicting treatment response of transarterial chemoembolization in hepatocellular carcinoma. BMC Cancer. (2023) 23:1026. doi: 10.1186/s12885-023-11491-0 37875815 PMC10594790

[30] Wang F Cheng M Du B Li J Li L Huang W . Predicting microvascular invasion in small (≤ 5 cm) hepatocellular carcinomas using radiomics-based peritumoral analysis. Insights into Imaging. (2024) 15(2024):90. doi: 10.1186/s13244-024-01649-0 38530498 PMC10965872

[31] Zhao Y Wang N Wu J Zhang Q Lin T Yao Y . Radiomics analysis based on contrast-enhanced MRI for prediction of therapeutic response to transarterial chemoembolization in hepatocellular carcinoma. Front Oncol. (2021) 11:582788. doi: 10.3389/fonc.2021.582788 33868988 PMC8045706

[32] Song W Yu X Guo D Liu H Tang Z Liu X . MRI-based radiomics: Associations with the recurrence-free survival of patients with hepatocellular carcinoma treated with conventional transcatheter arterial chemoembolization. J Magn Reson Imaging. (2020) 52:461–73. doi: 10.1002/jmri.26977 31675174

[33] Guo Z Zhong N Xu X Zhang Y Luo X Zhu H . Prediction of hepatocellular carcinoma response to transcatheter arterial chemoembolization: A real-world study based on non-contrast computed tomography radiomics and general image features. J Hepatocell Carcinoma. (2021) 8:773–82. doi: 10.2147/JHC.S316117 34277508 PMC8277455

[34] Piscaglia F Ogasawara S . Patient selection for transarterial chemoembolization in hepatocellular carcinoma: Importance of benefit/risk assessment. Liver Cancer. (2018) 7:104–19. doi: 10.1159/000485471 29662837 PMC5892363

[35] Wang S Wang S Liu X Wu Y Jiang C Luo Y . Habitat-based radiomics enhances the ability to predict lymphovascular space invasion in cervical cancer: a multi-center study. Front Oncol. (2023) 13:1252074. doi: 10.3389/fonc.2023.1252074 37954078 PMC10637586

[36] Wu J Cao G Sun X Lee J Rubin DL Napel S . Intratumoral spatial heterogeneity at perfusion MR imaging predicts recurrence-free survival in locally advanced breast cancer treated with neoadjuvant chemotherapy. Radiology. (2018) 288:26–35. doi: 10.1148/radiol.2018172462 29714680 PMC6029132

[37] Zhang Y Yang C Sheng R Dai Y Zeng M . Predicting the recurrence of hepatocellular carcinoma (≤ 5 cm) after resection surgery with promising risk factors: habitat fraction of tumor and its peritumoral micro-environment. Radiol Med. (2023) 128:1181–91. doi: 10.1007/s11547-023-01695-6 37597123

[38] Xue B-H Chen S-L Lan J-P Wang L-L Xie J-G Zheng X-W . Explainable PET-based habitat and peritumoral machine learning model for predicting progression-free survival in clinical stage IA pure-solid non-small cell lung cancer: A two-center study. Acad Radiol. (2025) 32(6):3687–98. doi: 10.1016/j.acra.2024.12.038 39757063

[39] Huang Z Huang W Huang W Jiang L Zheng Y Pan Y . Decision fusion model for predicting microvascular invasion in hepatocellular carcinoma based on multi-MR habitat imaging and machine-learning classifiers. Acad Radiol. (2023) 32(4):1971–80. doi: 10.1016/j.acra.2024.10.007 39472207

[40] Aerts HJWL Rios Velazquez E Leijenaar RTH Parmar C Grossmann P Carvalho S . Decoding tumour phenotype by noninvasive imaging using a quantitative radiomics approach. Nat Commun. (2014) 5:4006. doi: 10.1038/ncomms5006 24892406 PMC4059926

[41] Daginawala N Li B Buch K Yu HS Tischler B Qureshi MM . Using texture analyses of contrast enhanced CT to assess hepatic fibrosis. Eur J Radiol. (2016) 85:511–7. doi: 10.1016/j.ejrad.2015.12.009 26860661

[42] Mannelli L Kim S Hajdu CH Babb JS Clark TW Taouli B . Assessment of tumor necrosis of hepatocellular carcinoma after chemoembolization: diffusion-weighted and contrast-enhanced MRI with histopathologic correlation of the explanted liver. AJR Am J Roentgenol. (2009) 193:1044–52. doi: 10.2214/AJR.08.1461 19770328

[43] Kwan SW Fidelman N Ma E Kerlan RK Yao FY . Imaging predictors of the response to transarterial chemoembolization in patients with hepatocellular carcinoma: a radiological-pathological correlation. Liver Transplant. (2012) 18:Liver Transplant. doi: 10.1002/lt.23413 22344899 PMC4161220

[44] Meyer H-J Leonhardi J Höhn AK Pappisch J Wirtz H Denecke T . CT texture analysis of pulmonary neuroendocrine tumors-associations with tumor grading and proliferation. J Clin Med. (2021) 10:5571. doi: 10.3390/jcm10235571 34884272 PMC8658090

[45] Li Z Chen L Song Y Dai G Duan L Luo Y . Predictive value of magnetic resonance imaging radiomics-based machine learning for disease progression in patients with high-grade glioma. Quant Imaging Med Surg. (2023) 13:224–36. doi: 10.21037/qims-22-459 36620140 PMC9816734

[46] Liu L Zhao L Jing Y Li D Linghu H Wang H . Exploring a multiparameter MRI-based radiomics approach to predict tumor proliferation status of serous ovarian carcinoma. Insights Imaging. (2024) 15:74. doi: 10.1186/s13244-024-01634-7 38499907 PMC10948697

[47] Tabassum M Al Suman A Suero Molina E Pan E Di Ieva A Liu S . Radiomics and machine learning in brain tumors and their habitat: A systematic review. Cancers (Basel). (2023) 15. doi: 10.3390/cancers15153845 37568660 PMC10417709

[48] Huang YQ Liang HY Yang ZX Ding Y Zeng MS Rao SX . Value of MR histogram analyses for prediction of microvascular invasion of hepatocellular carcinoma. Med (Baltimore). (2016) 95:e4034. doi: 10.1097/MD.0000000000004034 27368028 PMC4937942

[49] Huang Y Qian H . Advancing hepatocellular carcinoma management through peritumoral radiomics: Enhancing diagnosis, treatment, and prognosis. J Hepatocell Carcinoma. (2024) 11:2159–68. doi: 10.2147/JHC.S493227 39525830 PMC11546143

[50] He X Zhou J Ye S Cheng Q Miao H Xu N . Differences in tumour heterogeneity based on dynamic contrast-enhanced MRI between tumour and peritumoural stroma for predicting Ki-67 status of invasive ductal carcinoma. Clin Radiol. (2021) 76:470.e13–470.e22. doi: 10.1016/j.crad.2020.12.008 33648758

[51] Luo H Zhao S Yang W Chen Z Li Y Zhou P . Preoperative prediction of extensive intraductal component in invasive breast cancer based on intra- and peri-tumoral heterogeneity in high-resolution ultrafast DCE-MRI. Sci Rep. (2024) 14:17396. doi: 10.1038/s41598-024-68601-6 39075278 PMC11286762

[52] Wu P-Q Guo F-L Wang J Gao Y Feng S-T Chen S-L . Development and validation of a dynamic contrast-enhanced magnetic resonance imaging-based habitat and peritumoral radiomic model to predict axillary lymph node metastasis in patients with breast cancer: a retrospective study. Quant Imaging Med Surg. (2024) 14:8211–26. doi: 10.21037/qims-24-558 39698668 PMC11651946

[53] Liu W Wang W Guo M Zhang H . Tumor habitat and peritumoral region evolution-based imaging features to assess risk categorization of thymomas. Clin Radiol. (2024) 79:e1117–25. doi: 10.1016/j.crad.2024.05.010 38862335

[54] Johnson PJ . The role of serum alpha-fetoprotein estimation in the diagnosis and management of hepatocellular carcinoma. Clin Liver Dis. (2001) 5:145–59. doi: 10.1016/s1089-3261(05)70158-6 11218912

[55] Tian M Zhang X Huang G Fan W Li J Zhang Y . Alpha-fetoprotein assessment for hepatocellular carcinoma after transarterial chemoembolization. Abdom Radiol (NY). (2019) 44:3304–11. doi: 10.1007/s00261-019-02116-x 31250181

[56] Lee YK Kim SU Kim DY Ahn SH Lee KH Lee DY . Prognostic value of α-fetoprotein and des-γ-carboxy prothrombin responses in patients with hepatocellular carcinoma treated with transarterial chemoembolization. BMC Cancer. (2013) 13:5. doi: 10.1186/1471-2407-13-5 23282286 PMC3545962

[57] Takayasu K Arii S Ikai I Omata M Okita K Ichida T . Prospective cohort study of transarterial chemoembolization for unresectable hepatocellular carcinoma in 8510 patients. Gastroenterology. (2006) 131:461–9. doi: 10.1053/j.gastro.2006.05.021 16890600

[58] Liu S Li H Guo L Zhang B Zhou B Zhang W . Tumor size affects efficacy of adjuvant transarterial chemoembolization in patients with hepatocellular carcinoma and microvascular invasion. Oncologist. (2019) 24:513–20. doi: 10.1634/theoncologist.2018-0305 30552155 PMC6459238

[59] Muñoz NM Minhaj AA Maldonado KL Kingsley CV Cortes AC Taghavi H . Comparison of dynamic contrast-enhanced magnetic resonance imaging and contrast-enhanced ultrasound for evaluation of the effects of sorafenib in a rat model of hepatocellular carcinoma. Magn Reson Imaging. (2019) 57:156–64. doi: 10.1016/j.mri.2018.11.012 30465870

[60] Lee J Steinmann A Ding Y Lee H Owens C Wang J . Radiomics feature robustness as measured using an MRI phantom. Sci Rep. (2021) 11:3973. doi: 10.1038/s41598-021-83593-3 33597610 PMC7889870

[61] Bai S Yang P Wei Y Wang J Lu C Xia Y . Development and validation of prognostic dynamic nomograms for hepatitis B virus-related hepatocellular carcinoma with microvascular invasion after curative resection. Front Oncol. (2023) 13. doi: 10.3389/fonc.2023.1166327 37152055 PMC10154689

[62] Austin PC . An introduction to propensity score methods for reducing the effects of confounding in observational studies. Multivar Behav Res. (2011) 46:399–424. doi: 10.1080/00273171.2011.568786 21818162 PMC3144483

