# Supplementary material for: Intratumoral habitat combined with peritumoral radiomics to improve the predictive performance of initial TACE efficacy: a multicenter retrospective study
Source: Front Oncol. 2026 Oct 14;16:1772206. doi: 10.3389/fonc.2026.1772206 (PMC13732582; doi:10.3389/fonc.2026.1772206)
Supplement: Supplementary file 1 [file SupplementaryFile1.docx]

**Supplementary Data**

S1. Magnetic resonance imaging sequences and parameters

S2. Detailed description of TACE procedure

S3. Radscore of the intratumoral habitat and peritumoral radiomics

**S1. Magnetic resonance imaging sequences and parameters**

| **Sequence** | **TR/ms** | **TE/ms** | **ST/mm** | **Spacing/mm** | **Matrix Size** | **FOV/mm** | **Fat suppression** |
| --- | --- | --- | --- | --- | --- | --- | --- |
| **Siemens Avanto 1.5Tesla (32-channel body array coil)** | | | | | | | |
| **Contrast-enhanced imaging with T1-weighted 3D GRE** | 4.7 | 2.4 | 4 | 1.8 | 220×320 | 380 | Yes |
| **T1-weighted imaging** | 7.7 | 4.7 | 4 | 1.8 | 220×320 | 380 | No |
| **T2-weighted imaging 2D FSE** | 3062 | 78 | 6.5 | 7.8 | 240×320 | 380 | Yes |
| **Diffusion-weighted imaging** | 5300 | 75 | 6 | 7.2 | 156×192 | 380 | Yes |
| **GE medical system discovery MR750 3.0 Tesla (32-channel body array coil)** | | | | | | | |
| **Contrast-enhanced imaging with T1-weighted 3D GRE** | 3.7 | 1.7 | 5 | 2.5 | 512×512 | 380 | Yes |
| **T1-weighted imaging** | 3.7 | 1.6 | 5 | 2.5 | 400×360 | 380 | No |
| **T2-weighted imaging 2D FSE** | 5000 | 76.1 | 6 | 7 | 512×512 | 380 | Yes |
| **Diffusion-weighted imaging** | 7500 | 54.4 | 6 | 7 | 256×256 | 380 | Yes |
| **Siemens Verio 3.0 Tesla (32-channel body array coil)** | | | | | | | |
| **Contrast-enhanced imaging with T1-weighted 3D GRE** | 4.0 | 1.4 | 3 | 1.5 | 240×320 | 360 | Yes |
| **T1-weighted imaging** | 5.4 | 2.4 | 3 | 1.5 | 240×320 | 360 | No |
| **T2-weighted imaging 2D FSE** | 5116.5 | 78 | 6 | 7.8 | 240×320 | 360 | Yes |
| **Diffusion-weighted imaging** | 4735.7 | 68 | 6 | 7.8 | 200×280 | 360 | Yes |
| **Note: TR = repetition time; TE = echo time; ST = section thickness; FOV = field of view** | | | | | | | |

**S2. Detailed description of TACE procedure**

The procedure was performed using the Seldinger technique to percutaneously puncture the femoral or radial artery, followed by insertion of a 5F-RH catheter sheath (Terumo, Tokyo, Japan). Subsequently, angiography of the superior mesenteric artery and abdominal aorta was performed to show the origin of the hepatic artery and its branches supplying the tumor. Ultraselection of tumor blood vessels was performed using a microcatheter (Merit Maestro, USA) under the guidance of a microintroducer (FATHOM, Boston Scientific, USA). For subsegment TACE, the emulsion contains 20~60 mg of doxorubicin (Actavis, Dublin, Ireland), 100~200 mg of oxaliplatin (Sichuan Huiyu Pharmaceutical, China), 40~100 mg of epirubicin (Adaison, Hanhui Pharmaceutical Co., Ltd., China), 500~1000 mg of 5-fluorouracil (Taijiben, Xin'an Pharmaceutical, China), and 0.5-15.0 mL of iodide (Youliying, Jiangsu Hengrui Pharmaceutical Co., Ltd., China). Alternatively, combine DEB particles with a diameter of 100 ~300 μm (CB, Jiangsu Hengrui Pharmaceutical Co., LTD., China) or 5% sodium bicarbonate (sodium bicarbonate). Then, use a 1 mm² gelatin sponge (CB; Jiangsu Hengrui Pharmaceutical Co., Ltd., China) or a coil (Boston Scientific, USA) to embolize the tumor's blood supply artery. The amount of iodized oil was determined according to the size of the tumor and the status of liver function. The interventional physician tried to embolize all tumors. The success of embolization was defined by a decrease in arterial blood flow to the tumor's blood supply or a reduction or disappearance of tumor staining in all targets.

**S3. Radscore of the intratumoral habitat and peritumoral radiomics**

(a) Intratumoral radscore:

0.023373814367244655*wavelet-HLL_glszm_SmallAreaHighGrayLevelEmphasis_A+0.19286943727837186*wavelet-HLH_glszm_SmallAreaLowGrayLevelEmphasis_A+0.01875695002425222*log-sigma-4-0-mm-3D_glszm_SizeZoneNonUniformity_PVP+0.1112670342005763*log-sigma-4-0-mm-3D_glszm_SmallAreaEmphasis_PVP+1.4855679922694842*wavelet-HLL_glcm_Imc1_PVP+0.008505477144035337*wavelet-HLH_glszm_SmallAreaLowGrayLevelEmphasis_PVP-0.11013791666876267*wavelet-HHH_firstorder_Skewness_PVP+0.07887710421215598*wavelet-LLL_glszm_SmallAreaEmphasis_PVP+0.00045520603044187156*wavelet-LLL_glszm_SmallAreaLowGrayLevelEmphasis_PVP+4.2876371295804084e-08*wavelet-LLL_glszm_ZoneVariance_PVP+0.003266631162060317*original_gldm_DependenceVariance_V+0.22524108222085734*log-sigma-3-0-mm-3D_glszm_SmallAreaLowGrayLevelEmphasis_V+0.23178760143980673*wavelet-LHL_glszm_SmallAreaEmphasis_V+0.03515911071891396*wavelet-LHL_glszm_SmallAreaLowGrayLevelEmphasis_V-0.023301943232420824*wavelet-LHH_glszm_SizeZoneNonUniformityNormalized_V+ 0.22462349288771397

(b) Peritumoral radscore:

0.13808590510445168*log-sigma-3-0-mm-3D_glcm_Imc1_A_Peritumor+0.12444342364871896*wavelet-LLH_gldm_SmallDependenceLowGrayLevelEmphasis_A_Peritumor-0.05644995227483594*wavelet-LHL_glcm_Imc2_A_Peritumor-0.0527476051926549*wavelet-LHH_glszm_GrayLevelNonUniformityNormalized_A_Peritumor+4.6475169391947615e-15*wavelet-LHH_glszm_GrayLevelVariance_A_Peritumor+0.03881649362546659*wavelet-HLH_glszm_SmallAreaLowGrayLevelEmphasis_A_Peritumor+0.0028836361274641345*wavelet-LLL_firstorder_Skewness_A_Peritumor+0.01640823400800846*log-sigma-3-0-mm-3D_glszm_SmallAreaEmphasis_PVP_Peritumor+0.022963167158294315*log-sigma-4-0-mm-3D_glszm_SizeZoneNonUniformity_PVP_Peritumor+0.042891335166576006*log-sigma-4-0-mm-3D_glszm_SmallAreaEmphasis_PVP_Peritumor+0.02918866152041461*wavelet-LHH_glrlm_ShortRunLowGrayLevelEmphasis_PVP_Peritumor+0.027484876783011487*wavelet-LHH_glszm_HighGrayLevelZoneEmphasis_PVP_Peritumor+-3.682239698340106e-15*wavelet-LHH_glszm_LowGrayLevelZoneEmphasis_PVP_Peritumor-0.018440392941689088*wavelet-HLL_glcm_Imc2_PVP_Peritumor+0.11300558502983846*wavelet-HLH_glrlm_ShortRunHighGrayLevelEmphasis_PVP_Peritumor+0.0347174341594816*wavelet-HLH_glszm_SmallAreaLowGrayLevelEmphasis_PVP_Peritumor+0.05367382673108322*wavelet-HHL_firstorder_Median_PVP_Peritumor+0.09234138058837242*wavelet-LLL_firstorder_Skewness_PVP_Peritumor+0.06868423886692442*wavelet-LLL_glszm_ZoneVariance_PVP_Peritumor+0.04625627335586426*wavelet-LHL_glszm_SmallAreaEmphasis_V_Peritumor-0.0832145232371457*wavelet-LHH_glrlm_GrayLevelNonUniformityNormalized_V_Peritumor+1.2648678400969036e-13*wavelet-LHH_glrlm_GrayLevelVariance_V_Peritumor+0.07863412896211401*wavelet-LHH_glrlm_ShortRunLowGrayLevelEmphasis_V_Peritumor+0.03935766217413235*wavelet-HLL_glcm_Imc1_V_Peritumor+0.43055555555555486
